# Choosing MUSE: Validation of a Low-Cost, Portable EEG System for ERP Research

**DOI:** 10.3389/fnins.2017.00109

**Published:** 2017-03-10

**Authors:** Olave E. Krigolson, Chad C. Williams, Angela Norton, Cameron D. Hassall, Francisco L. Colino

**Affiliations:** Neuroeconomics Laboratory, Centre for Biomedical Research, University of VictoriaVictoria, BC, Canada

**Keywords:** EEG, ERP, portable electronics, cognitive science, executive function

## Abstract

In recent years there has been an increase in the number of portable low-cost electroencephalographic (EEG) systems available to researchers. However, to date the validation of the use of low-cost EEG systems has focused on continuous recording of EEG data and/or the replication of large system EEG setups reliant on event-markers to afford examination of event-related brain potentials (ERP). Here, we demonstrate that it is possible to conduct ERP research without being reliant on event markers using a portable MUSE EEG system and a single computer. Specifically, we report the results of two experiments using data collected with the MUSE EEG system—one using the well-known visual oddball paradigm and the other using a standard reward-learning task. Our results demonstrate that we could observe and quantify the N200 and P300 ERP components in the visual oddball task and the reward positivity (the mirror opposite component to the feedback-related negativity) in the reward-learning task. Specifically, single sample *t*-tests of component existence (all *p*'s < 0.05), computation of Bayesian credible intervals, and 95% confidence intervals all statistically verified the existence of the N200, P300, and reward positivity in all analyses. We provide with this research paper an open source website with all the instructions, methods, and software to replicate our findings and to provide researchers with an easy way to use the MUSE EEG system for ERP research. Importantly, our work highlights that with a single computer and a portable EEG system such as the MUSE one can conduct ERP research with ease thus greatly extending the possible use of the ERP methodology to a variety of novel contexts.

## Introduction

In recent years there has been an almost explosive growth of low-cost (i.e., less than $500 USD) electroencephalographic (EEG) recording systems. While most of the systems on the market offer software developer kits allowing scientists to access the raw data for research purposes—only a small amount of work has been done to validate the effectiveness of these systems for event-related brain potential (ERP) research (e.g., Debener et al., 2012; Badcock et al., 2013; Duvinage et al., 2013; Gramann et al., 2014; Wascher et al., 2014; Badcock et al., 2015; Maskeliunas et al., 2016; Kuziek et al., 2017). Indeed, research to date has focused on the replication of a large array EEG setup in which event-markers are used to temporally synchronize the EEG data to events of interest (Debener et al., 2012; Vos et al., 2014) or temporal synchronization of time stamps between computers to determine when events occur (Wong et al., 2014). Further, the majority of portable EEG studies to date have relied on electrode caps or full coverage electrode arrays which also negates the use of these systems in certain environments and increases the difficulty of participant setup—something that at an inherent level takes away from the portability and ease of use of these systems.

From a technical perspective, the ERP methodology is difficult to implement in low-cost non-standard research grade equipment for several reasons. First and foremost, there is the issue of data quality and whether low-cost systems can deliver sampling rates (i.e., >= 250 Hz) and data quality (i.e., noise free, a small number of artifacts) conducive for traditional ERP analyses. Two other key issues that are a cause for concern relate to the issue of experimental timing: first, how one can “mark” the data for subsequent ERP analysis, and second, the issue of non-standard electrode locations for analysis—ERP components are typically associated with analysis of specific electrode locations.

When designing and implementing ERP experiments, it is of key importance that steps be taken to assure that the highest quality of data is recorded. Specifications to enhance the quality of data collection were specifically highlighted in the seminal Picton paper (Picton et al., 2000; see also Luck, 2014) in which issues such as electrode type, electrode quality (e.g., Coles et al., 1986; Kutas, 1997), the minimum number of electrodes necessary for meaningful interpretation (e.g., Srinivasan et al., 1998), and the capabilities of the amplifier (e.g., Cadwell and Villarreal, 1999) were stated to specifically increase data quality and thus the ability to draw meaningful conclusions from EEG/ERP data. Amplifier characteristic such as the number of bits available for the converter (8 minimum), the gain of the amplifier, and the common-mode rejection ratio were also stated in the Picton paper (Picton et al., 2000) to have minimum values necessary to achieve sufficient EEG data quality. As such, an obvious concern with the use of low-cost EEG systems is whether the actual hardware meets the standards needed to achieve sufficient EEG data quality (e.g., quality of the electrodes on a low-cost EEG system). Indeed, if the minimum standards set out in the Picton paper (and in other sources) are not met in a low-cost EEG system it suggests that these systems will not be able to provide EEG data of sufficient quality for meaningful interpretation. While we agree that all the concerns that have bene outlined in the Picton paper (and other sources) are valid, a more meaningful test of data quality is rather straightforward—collect data from a low-cost system and directly determine whether said EEG system can provide data that reliably results in visible and statistically quantifiable ERP components. Indeed, the existing studies that have examined the efficacy of portable, low cost EEG systems for ERP research suggest that it is possible to collect data of sufficient quality for ERP analyses (e.g., Debener et al., 2012; Vos et al., 2014).

Another key issue that occurs when using a low-cost EEG system for ERP research relates to the issue of event timing. Typically, in an ERP paradigm an event marker is sent from a stimulus computer to a recording computer via a parallel or TTL cable to “mark” the data. Importantly, marking the data in this manner affords the ability to precisely extract epochs of data centered on the onset of events of interest and thus allows the researcher to create event-related average waveforms for subsequent analysis (Coles et al., 1986; Luck, 2014). To date, studies using portable EEG systems have attempted to mirror this procedure (Debener et al., 2012; Vos et al., 2014) or have attempted to use temporal synchronization to obtain precise event timing (Wong et al., 2014). Here, we take a different approach to event-timing and directly record EEG data after each event thus negating the need to mark the continuous EEG data. While we appreciate there is temporal jitter in the data due to this approach in addition to timing variability due to Bluetooth communication (see methods) these temporal inconsistencies are Gaussian in nature and should average out during data analysis to yield reliable ERP components.

A final issue that relates to the use of portable, low cost EEG systems is the potential that electrode channels are not available in locations that are associated with specific ERP components. Typically, researchers seek to analyze specific electrode channels for specific ERP components—channels where the ERP component is maximal and has been reported before (Rugg and Coles, 1995; Luck, 2014). Here, we hypothesized that ERPs could be recorded with a MUSE system in a dramatically more efficient way at a fraction of the cost making the trade-off between electrode location and ease of use worthwhile. Indeed, after an a priori analysis of pilot data, we decided to simply analyze ERP components at non-standard locations. For example, while the P300 ERP component is typically maximal at posterior locations on the midline we did not have an electrode(s) at this location and as such we were forced to analyze the P300 component at electrode locations that we did have (here, electrodes TP9 and TP10). While obviously our solutions to this issue is not ideal, the purpose of the present research was to demonstrate that a low-cost EEG system could be used to conduct ERP research and thus we “worked with what we had.”

In the present experiment we specifically sought to test whether or not the MUSE EEG system (InterAxon Inc.) could be used to quickly collect EEG data that would yield observable and quantifiable ERP components without the use of event-markers—specifically the N200, P300, and reward positivity (also known as the feedback related negativity: see Proudfit, 2015 for review). The N200 and P300 ERP components have been shown to be sensitive to stimulus frequency and are typically evoked via the oddball paradigm (e.g., Squires et al., 1975). The reward positivity is typically defined as a difference in between feedback locked ERP waveforms indicating wins and losses or correct and incorrect trials and is thought to reflect a reinforcement learning system within the medial-frontal cortex (e.g., Holroyd and Coles, 2002). All three components were chosen as they are typically quite large (in terms of μV effect size) and between the three of them index a wide range of cognitive and perceptual phenomena. To validate the effectiveness of the MUSE system for ERP research, we collected MUSE EEG data while 60 participants performed both an oddball and a reward-learning task on a laptop computer. For comparison purposes, we randomly selected the data from a matched number of participants who had performed these same two tasks while EEG data was recorded on a Brain Vision ActiChamp system with a standard 10–20 electrode configuration.

Our hypothesis was simple—we predicted that we would be able to see and quantify the N200, P300, and reward positivity in the EEG data we collected with the MUSE system without having to be reliant on event-markers. Further, we predicted that a comparison of the “MUSE components” with ERP components observed in a reduced analysis pipeline (see below) from our large array system would reveal that the two sets of components from different EEG systems were the same. Finally, we note here that one of the principle reasons we sought to test the MUSE EEG system in this manner was to develop a portable and efficient method of measuring the aforementioned ERP components for field and/or clinical research. As such, we deliberately collected a minimal amount of data—our goal, which we accomplished, was for EEG setup and data collection to be finished in under 10 min.

## Methods

### Participants

Undergraduate students (*n* = 60; MUSE group, 34 female, mean age: 21) from the University of Victoria participated in the experiment using the MUSE portable EEG system (SCR_014418). For comparison purposes, we randomly selected 60 undergraduate participants (Standard/Reduced group: 34 females, mean age: 20.9 years) from the University of Victoria from an existing long-term project in our laboratory as a comparison group for analysis purposes. Participants in this “standard/reduced group” had performed the same two experimental tasks as the MUSE group but instead their EEG data was collected using a Brain Vision ActiChamp System with a standard 10-10 electrode configuration and a typical EEG set-up (stimulus machine, recording machine, etc.). All participants had normal or corrected-to-normal vision, no known neurological impairments, volunteered for extra course credit in a psychology course and provided written informed consent approved by the Human Research Ethics Board at the University of Victoria. The study followed ethical standards as prescribed in the 1964 Declaration of Helsinki.

### Apparatus and procedure

#### Standard group

**Figure 1:**
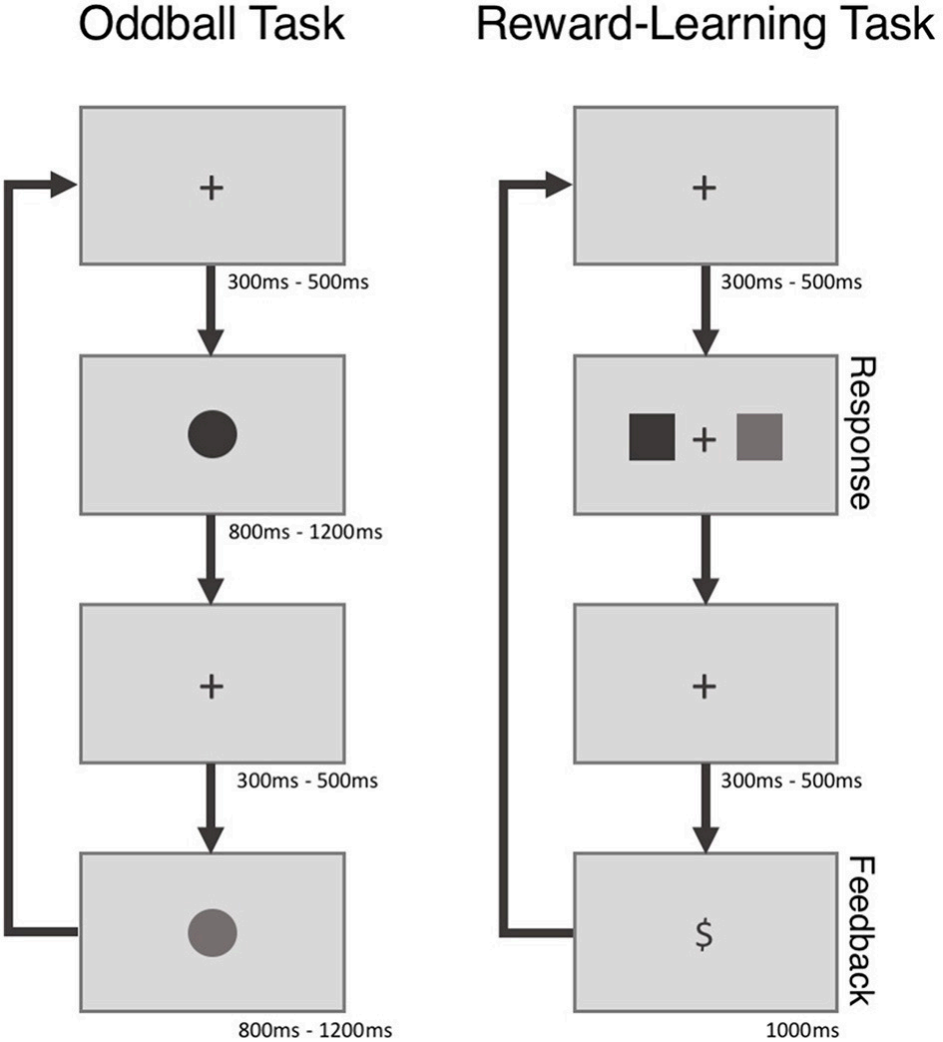
**The experimental trial time line for both tasks**.

Participants in the standard group were seated in a sound dampened room in front of a 19″ LCD computer monitor and used a standard USB mouse to complete the two experimental tasks - a visual oddball task and a reward-learning task—while EEG data were recorded via an ActiChamp system (see Figure 1 for a time line for both tasks—the tasks themselves are described below). The experimental tasks were coded in MATLAB programming environment (Version 8.6, Mathworks, Natick, U.S.A.) using the Psychophysics Toolbox extension (Brainard, 1997).

During performance of the oddball task participants saw a series of blue (MATLAB RGB value = [0 0 255]) and green (MATLAB RGB value = [0 255 0]) colored circles that appeared for 800–1,200 ms in the center of a dark gray screen (MATLAB RGB value = [108 108 108]). Prior to the onset of the first circle and in between the presentation of subsequent circles a black fixation cross was presented for 300 to 500 ms (MATLAB RGB value = [0 0 0]). Participants were not told that the frequency of the blue and green circled differed: the blue circles appeared less frequently (oddball: 25%) than the green circles (control: 75%) with the sequence order of presented circles being completely random. Participants were instructed to mentally count the number of blue circles (oddballs) within each block of trials. Participants completed 3 blocks of 40 trials during performance of the oddball task.

On each trial of the reward-learning task participants viewed a black fixation cross (MATLAB RGB value = [0 0 0]) for 300 to 500 ms that was followed by a blue and a green pair squares (MATLAB RGB values = [0 0 255] and [0 255 0], respectively). Participants were asked on each trial to select one of the two squares. Following the selection of a square, the black fixation cross (MATLAB RGB value = [0 0 0]) reappeared for 300 to 500 ms following which a reward stimulus in black (“$” for wins, “0” for losses) (MATLAB RGB values = [0 0 0]) was shown for 1,000 ms. Immediately following the offset of the feedback stimulus the next experimental trial began. The reward structure of the squares was such that selection of one of the square colors resulted in more frequent wins than the other (60 vs. 10% win/loss ratio). The location of each square (left, right) was randomly determined each trial and the win/loss percentage to color relationship remained constant for each block of trials. Participants completed 5 blocks of 20 trials and unique square colors were used for each block of trials.

#### MUSE group

The oddball and reward-learning tasks that participants performed in the MUSE group were identical except for a few minor differences. The tasks were performed in a quiet room on a 11″ MacBook Air laptop (Apple Inc., California, USA) with participants wearing a MUSE EEG headband. Importantly, although the monitor size was reduced, the stimuli sizes were adjusted to be the same as the standard task. Participants responded via the “a” and “l” keys on the laptop keyboard.

### Data acquisition

#### Standard group

EEG data in the standard group were recorded using Brain Vision Recorder software (Version 1.21, Brainproducts, GmbH, Munich, Germany) and 64 electrodes that were mounted in a fitted cap with a standard 10-10 layout (ActiCAP, Brainproducts GmbH, Munich, Germany: the specific cap layout is available here http://www.neuroeconlab.com/electrode-configuration.html). Electrodes on the cap were initially referenced to a common ground. On average, electrode impedances were kept below 20 kΩ. The EEG data were sampled at 500 Hz, amplified (ActiCHamp, Revision 2, Brainproducts GmbH, Munich, Germany), and filtered through an antialiasing low-pass filter of 8 kHz. To ensure temporal coincidence of event-markers with experimental stimuli a DATAPixx stimulus unit was used (VPixx, Vision Science Solutions, Quebec, Canada).

#### MUSE group

EEG data in the MUSE group were recorded from a MUSE EEG headband with research preset AD (500 Hz sampling rate, no onboard data processing: InteraXon, Ontario, Canada) (see http://developer.choosemuse.com/hardware-firmware/hardware-specifications for full technical specifications). The MUSE EEG system has electrodes located analogous to Fpz, AF7, AF8, TP9, and TP10 with electrode Fpz utilized as the reference electrode. Using the muse-io SDK we streamed data from the MUSE EEG system directly to MATLAB via the open sound control (OSC) protocol (see http://www.neuroeconlab.com/muse.html for all configuration, setup, and acquisition methods and software). In essence, following the presentation of each experimental stimulus of interest we directly sampled 1,000 ms of streaming data into MATLAB—subject to a small, varying inherent timing lag due to the Bluetooth connection (see http://developer.choosemuse.com/protocols/data-streaming-protocol). We tested the latency and variability of the latency of the Bluetooth EEG data stream by sending a series of 5000 TTL pulses into the MUSE auxiliary port from MATLAB and measuring the time it took for these pulses to “return” and be visible in the sampled EEG data. This test demonstrated a mean lag of 40 ms (±20 ms). It is important to note that this time includes the transmission time of the TTL pulse to the MUSE, the time back from MUSE system via Bluetooth, the conversion to an osc format via muse-io (the MUSE SDK software), and time needed to read the osc message stream into MATLAB. We also note here, however, this variability was in part due to a few samples (*n* < 10) with extreme latencies.

### Data processing

#### Standard analysis

Data were processed offline with Brain Vision Analyzer 2 software (Version 2.1.1, Brainproducts, GmbH, Munich, Germany) using methods we have previously employed (see http://www.neuroeconlab.com/data-analysis.html). First, excessively noisy or faulty electrodes were removed. The ongoing EEG data were re-referenced to an average mastoid and then filtered using a dual pass Butterworth filter with a passband of 0.1 Hz to 30 Hz in addition to a 60 Hz notch filter. Next, segments encompassing the onset of each event of interest (1,000 ms before to 2,000 ms after) were extracted from the continuous EEG. Following segmentation, independent component analysis was used to correct ocular artifacts (Delorme and Makeig, 2004; Luck, 2014). Data were reconstructed after the independent component analysis and any channels that were removed initially were interpolated using the method of spherical splines. New, shorter epochs were then constructed—from 200 ms before to 600 ms after the onset of each event of interest. In the oddball task, these events were the onset of the oddball and control circle stimuli; in the reward-learning task, these events were the onset of the win and loss feedback stimuli. Following this, all segments were baseline corrected using a 200 ms window preceding stimulus onset. Finally, all segments were submitted to an artifact rejection algorithm that marked and removed segments that had gradients of greater than 10 μV/ms and/or a 100 μV absolute within segment difference.

For each participant and event of interest, ERP waveforms were created by averaging the segmented EEG data for each electrode. Subsequently, a difference waveform was created by subtracting the control waveforms from the oddball waveforms in the oddball task and the loss waveforms from the win waveforms in the reward-learning task. For each conditional and difference waveform, a grand average waveform was created by averaging corresponding ERPs across all participants. ERP components of interest were quantified by first identifying the time point of maximal deflection from 0 μV on the appropriate grand average difference waveform in the time range of the component at the channel where this deflection was maximal (N200: 236 ms; P300: 397 ms; reward positivity: 301 ms)—with the channel also being verified to be inline with previous literature (N200: Pz; P300: Pz, reward positivity: FCz). All peaks were then quantified on an individual basis by taking the mean voltage ±25 ms of the respective time points on the respective channels for each participant.

#### Reduced analysis

To afford a better ERP component comparison with the MUSE group a second “reduced analysis” was conducted on the data from the standard group again with Brain Vision Analyzer 2 software. First, all channels except for Fpz, AF7, AF8, TP9, and TP10 were removed from all subsequent analysis steps. Next, the continuous EEG data were re-referenced to electrode Fpz electrode and this electrode was then also removed to replicate the data we recorded from the MUSE EEG system. Data was then left referenced to FPz for the analysis of oddball data or re-referenced to the average of electrodes TP9 and TP10 for the analysis of the reward-learning data. Data were then filtered with a dual pass Butterworth filter with a passband of 0.1 Hz to 15 Hz^1^ in addition to a 60 Hz notch filter. The data was then segmented from the onset of the stimulus of interest to 600 ms after. Next, a baseline correction was applied to each segment using a window from 0 to 50 ms—a window that was chosen as we did not record EEG data prior to stimulus onset with the MUSE system. An artifact rejection algorithm was then implemented; as a result of this procedure segments that had gradients of greater than 10 μV/ms and/or an absolute difference of more than 100 μV were discarded. The segmented data were then separated by experimental condition for each of the two tasks (oddball task: oddball, control; reward-learning task: win, loss). For the oddball task electrodes TP9 and TP10 were then pooled and event-related potential averages were created for each condition (oddball, control). For the reward-learning task electrodes AF7 and AF8 were then pooled and event-related potential averages were created for each condition (win, loss). Finally, a difference waveform was created by subtracting the control waveforms from the oddball waveforms in the oddball task and the loss waveforms from the win waveforms in the reward-learning task. For each conditional and difference waveform, a grand average waveform was created by averaging corresponding ERPs across all participants. ERP components were extracted and quantified in the same manner as the standard analysis, however obviously enough only on the pooled channel. The grand average component peak times were 233 ms for the N200, 339 ms for the P300, and 293 ms for the reward positivity.

#### MUSE analysis

The MUSE EEG was processed identically to the reduced analysis above; however, first—we had to convert the MUSE data into a format suitable for analysis in Brain Vision Analzer (this software is available at http://www.neuroeconlab.com/muse-analysis.html). Following the analysis of the MUSE data, the ERP components of interest were quantified in a manner also identical to the reduced analysis. Due to the timing lag inherent with the Bluetooth connection, the MUSE grand average difference peak times lagged and thus were slightly different (N200: 260ms, P300: 381ms, reward positivity: 297 ms) from the reduced analysis peak times.

### Data analysis

For all analyses (standard, reduced, MUSE), the same statistical procedures were used. For each component (N200, P300, reward positivity) analyses were conducted on the mean peak amplitudes extracted from the difference waves. To confirm the differences between conditions of each component, we compared the mean peak difference data to zero using three statistical methods: 95% confidence intervals, *t*-tests (α = 0.05), and 95% highest density Bayesian credible intervals.

## Results

**Figure 2:**
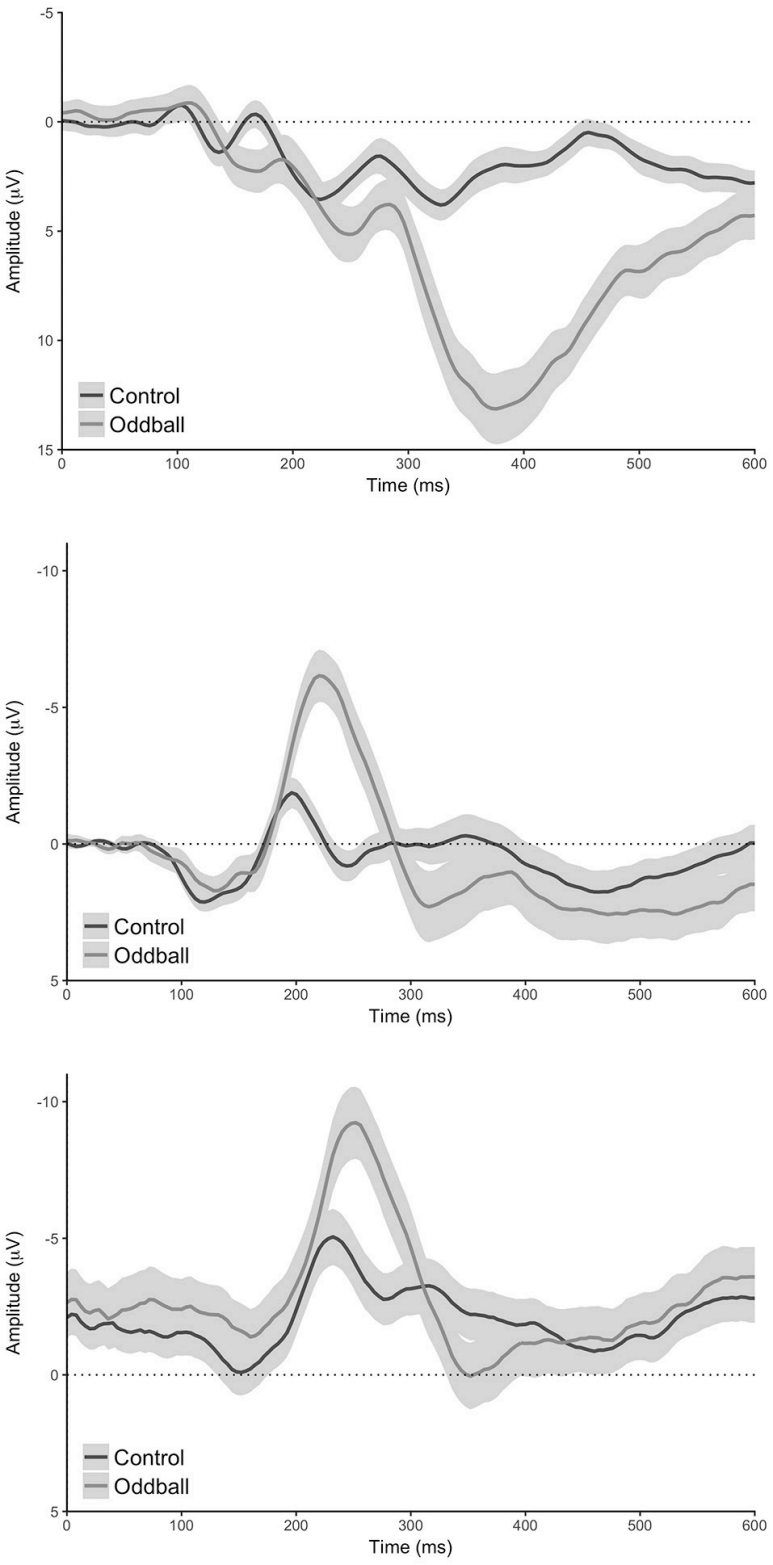
**Conditional waveforms for the oddball task. Top:** standard analysis (electrode Pz), **middle**: reduced analysis (pooled electrode TP9 & TP10), **bottom**: MUSE analysis (pooled electrode TP9 & TP10). Shaded regions reflect 95% confidence intervals around the grand average waveform.

**Figure 3:**
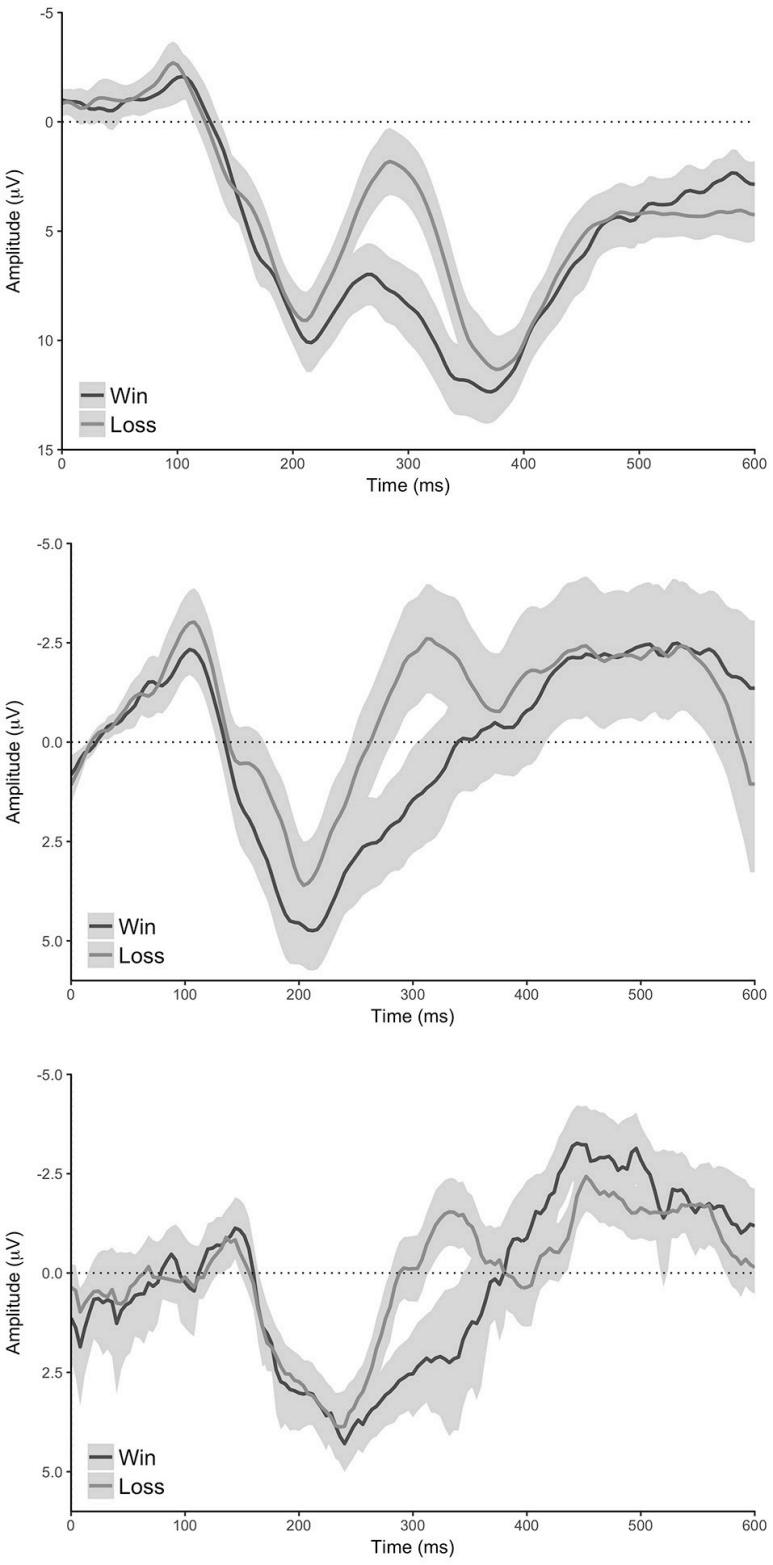
**Conditional waveforms of the reward learning task. Top:** standard analysis (electrode FCz), **middle**: reduced analysis (pooled electrode AF7 & AF8), **bottom**: MUSE analysis (pooled electrode AF7 & AF8). Shaded regions reflect 95% confidence intervals around the grand average waveform.

**Figure 4:**
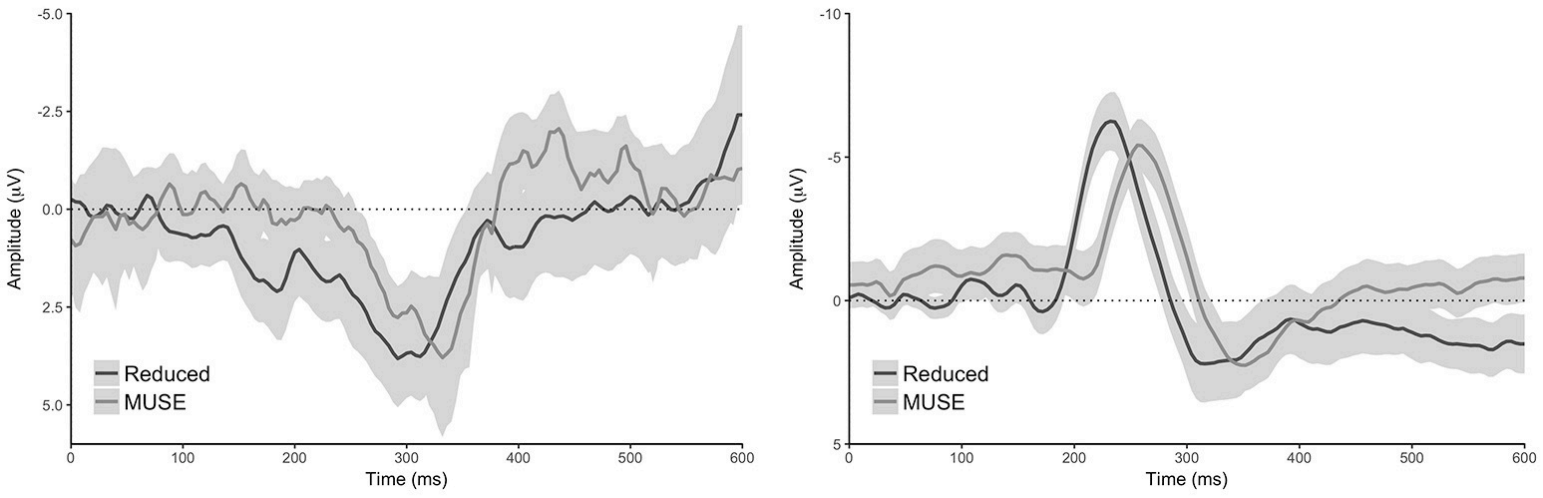
**Difference waveforms of the reduced and MUSE analysis for both tasks. Left:** oddball task, **right:** reward learning task. Difference waveforms were created by subtracting the control condition from the oddball condition for the oddball task, and the loss condition from the win condition for the decision making task. Shaded regions reflect 95% confidence intervals around the grand average waveform.

**Figure 5:**
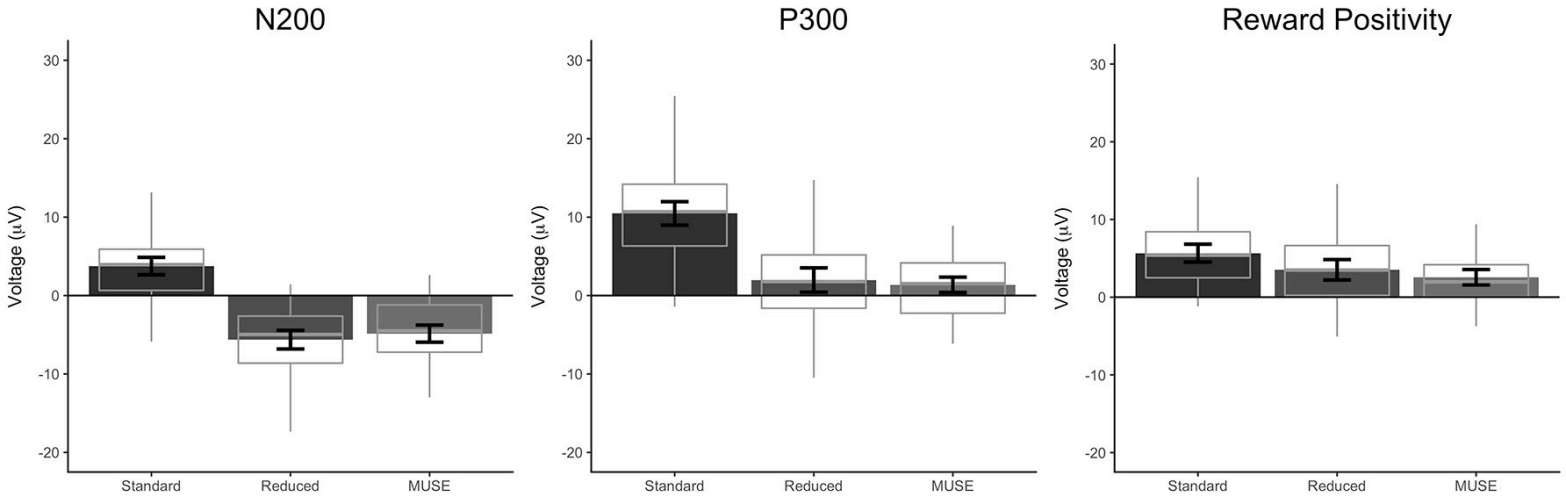
**Mean amplitudes with 95% confidence intervals of the N200 (left), P300 (middle), and reward positivity (right) for the standard, reduced, and MUSE analyses**. Mean amplitudes were calculated by averaging 25 ms surrounding the respective peaks.

Our analyses of the grand average difference waveforms revealed components with a timing consistent with the N200, P300, and reward positivity for all analyses (see Figures 2–4). Furthermore, all statistical tests determined that there was indeed a difference in all component peaks as a function of experimental condition for all analyses (see Figure 5 and Supplementary Figure 1).

### Oddball task: N200

Our analysis of the standard data revealed that oddball stimuli elicited a difference in the amplitude of the N200 relative to control stimuli in line with previous findings (M_d_ = 3.75 μV [2.64 μV 4.87 μV], *t*_(59)_ = 6.76, *p* < 0.0001, Bayesian HDI: μ = 3.72 μV [2.57 μV 4.83 μV]). Our reduced analysis demonstrated a similar, but reversed effect, relative to the standard analysis—oddball stimuli elicited a more positive voltage in the N200 time range than control stimuli (M_d_ = −5.63 μV [−6.81 μV −4.44 μV], *t*_(59)_ = −9.51, *p* < 0.0001, Bayesian HDI: μ = −5.57 μV [−6.76 μV −4.43 μV]). Importantly, our analysis of the MUSE data revealed a component that was identical to the N200 observed in the reduced analysis (M_d_ = −4.85 μV [−5.95 μV −3.76 μV], *t*_(59)_ = −8.89, *p* < 0.0001, Bayesian HDI: μ = −4.80 μV [−5.91 μV −3.69 μV]).

### Oddball task: P300

Our analysis of the standard data revealed that oddball stimuli elicited a difference in the amplitude of the P300 relative to control stimuli in line with previous findings (M_d_ = 10.47 μV [8.98 μV 11.97 μV], *t*_(59)_ = 14.00, *p* < 0.0001, Bayesian HDI: μ = 10.41 μV [8.87 μV 11.90 μV]). As with the N200, our reduced analysis also revealed a difference in the amplitude of the P300 between the oddball and control stimuli—and yet again it was reversed in polarity (M_d_ = 1.98 μV [0.43 μV 3.53 μV], *t*_(59)_ = 2.55, *p* = 0.0132, Bayesian HDI: μ = 1.91 μV [0.36 μV 3.48 μV]). Again, our analysis of the MUSE data revealed a P300 component that was for all practical purposes identical to the component we observed in the reduced analysis (M_d_ = 1.37 μV [0.39 μV 2.35 μV], *t*_(59)_ = 2.80, *p* = 0.0069, Bayesian HDI: μ = 1.36 μV [0.36 μV 2.36 μV]).

### Reward-learning task: the reward positivity

Our standard analysis revealed that the “win” stimuli in the reward-learning task differentially modulated the amplitude of the reward positivity relative to “loss” stimuli—a result in line with previous findings (M_d_ = 5.67 μV [4.51 μV 6.82 μV], *t*_(59)_ = 9.83, *p* < 0.0001, Bayesian HDI: μ = 5.58 μV [4.40 μV 6.75 μV]). The reduced analysis found an effect that was similar to the standard analysis (M_d_ = 3.52 μV [2.21 μV 4.84 μV], *t*_(59)_ = 5.36, *p* < 0.0001, Bayesian HDI: μ = 3.45 μV [2.14 μV 4.76 μV]), and the MUSE analysis revealed a similar effect to the reduced analysis (M_d_ = 2.57 μV [1.58 μV 3.57 μV], *t*_(59)_ = 5.19, *p* < 0.0001, Bayesian HDI: μ = 2.32 μV [1.38 μV 3.30 μV]).

### Additional analyses

**Figure 6:**
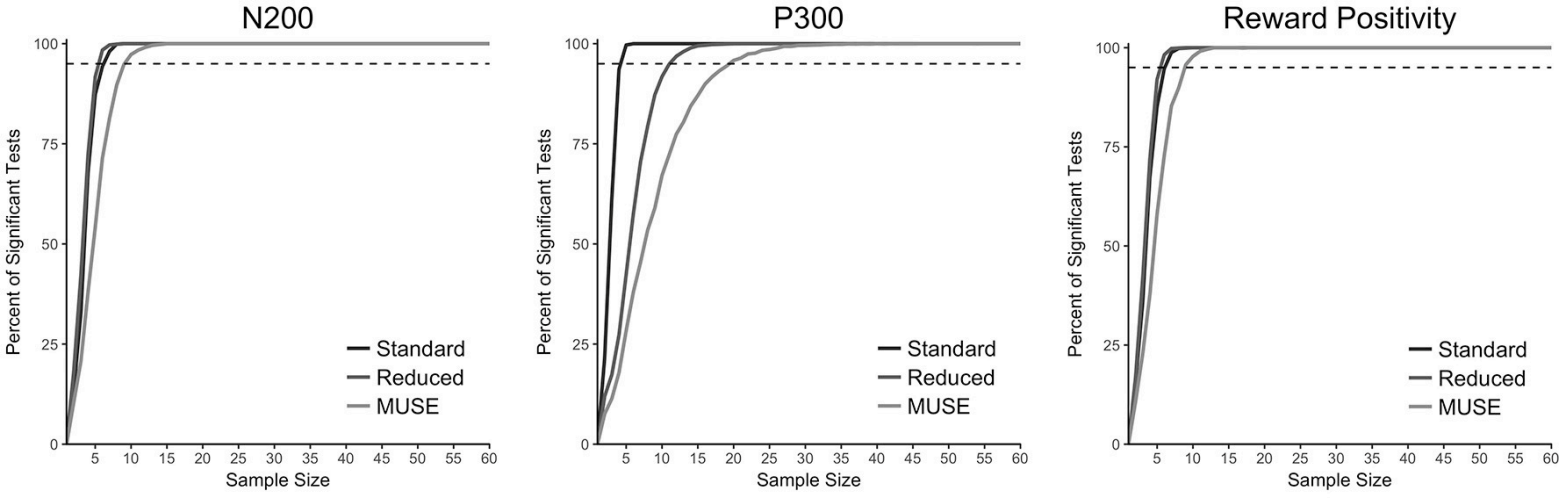
**Resampling analysis to test the minimum number of participants needed to achieve statistical significance reported as the percent of significan***t***-tests (***p*** < 0.05) out of 10,000**. The dashed horizontal line is placed at 95%.

To test the reliability of ERP data collection with MUSE, we also conducted a resampling analysis in which we computed the percentage of significant statistical tests for 10,000 samples of the data with sample sizes ranging from 2 to 60 for each of our two groups and three analysis procedures—standard, reduced, MUSE. The results of this analysis are presented in Figure 6 and demonstrate that for the N200 and reward positivity a sample size of 10 is more than sufficient to achieve reliable results. However, for the P300 component we found that for the reduced and MUSE groups this sample size was greater—perhaps due to a greater the attenuation of this ERP component at the electrode sites that we used to quantify it, TP9 and TP10.

## Discussion

The results presented here clearly demonstrate that the MUSE EEG system can be used to conduct event-related brain potential (ERP) research from a single computer without the use of event-markers. Specifically, in two separate tasks, a visual oddball task and a reward-learning task, we demonstrated that the data we collected with MUSE EEG system yielded the N200, P300, and reward positivity components. A resampling analysis implemented *post-hoc* (see Figure 6) clearly demonstrates as well that one can measure reliable ERP components with MUSE (especially the N200 and reward positivity) with a minimal number of participants. Further, we note there that the time to complete both experimental tasks—including EEG setup—was done, on average, in less than 10 min. For comparison purposes, one must consider the task completion time with our large array ActiChamp system. On average, two skilled research assistants took 35 min to affix an electrode cap, 64 electrodes, apply gel, and get electrode impedances within an acceptable range. While the task time remained the same, post experiment it took the two research assistants 15 min to clean up bringing the total testing time to 60 min, on average. As such, the setup time and testing time with the MUSE was approximately one sixth of the setup and testing time with our large array system. Other points to consider here are the fact that large array systems typically require two (or more) research assistants vs. one with our MUSE setup and cost considerably more approximately $75,000 vs. $250 for the MUSE system (see Table 1).

**Table 1:** **EEG System Comparison**.

	**Number of EEG channels**	**Number of AUX inputs**	**Sampling rate**	**Testing time^a^ (min)**	**Cost (USD)**
actiCHamp	32–160	8^b^	Up to 100 KHz	60	$77,100
Muse (2014 version)	5	2^c^	220 Hz or 500 Hz^d^	10	$249

a *These are average testing times for the current study, and include setup, task time, and cleanup*.

b *Additional sensors for GSR, EOG, EMG, ECG, respiration, acceleration, temperature, blood pulse, light (photosensor), or sound (microphone) may be used*.

c *EMG, ECG, or EEG electrodes may be attached via two micro-USB ports (one port for the 2016 version)*.

d *The 2016 version samples at 256 Hz*.

The data collected here provide further support for the use of low-cost, portable EEG systems such as the MUSE for field research (i.e., Debener et al., 2012). Our work however, further extends the portability and ease of use aspects of previous work given that we were able to collect two complete experiments (including setup) in 10 min, we were able to do this with a single, small laptop computer, and further we were able to move away from the traditional use of event-markers. To some extent, our approach replicated previous work (Vos et al., 2014; Wong et al., 2014) but our technique greatly improves the portability and ease of use of mobile ERP data collection. Our work here increases the ability of researchers to collect EEG data in clinical settings and out in the “real world.” Furthermore, given the low cost of the MUSE and its ease of use, the MUSE system affords the ability to collect large numbers of participants simultaneously with relative ease. With that said, there are some key concerns that researchers need to be aware of when using a portable EEG protocol such as the one presented here.

### Data quality

Perhaps the biggest difficulty that was faced in the present study was setting up participants with the MUSE headband for data collection. Indeed, during our pilot work our research assistants initially had trouble obtaining a sufficient level of data quality from the MUSE system. But, for the most part, this lost data was due to an inability of the research assistants to achieve a sufficient connection between the EEG headband and the respective electrode scalp locations during setup—which we restricted on purpose to 5 min after which we began the experiment anyways. One thing we note here, was that certain head shapes, head sizes, and hair styles made data collection difficult—a factor that needs to be considered when designing ERP studies with portable EEG systems such as the MUSE. With that said, once our research assistants gained a sufficient level of experience with the MUSE system our ratio of lost participants dropped to one out of twenty, a number that is in line with ERP studies done with large array EEG systems. Further, a quick inspection of the MUSE website reveals tutorials and support videos that will help future researchers learn to quickly and properly put a MUSE headset on participants.

To improve our ability to assess data quality during setup and the course of the experiment, we wrote a MATLAB script that showed the raw EEG in real-time to the research team. Importantly, our software also showed the variance of the EEG signal for each channel per second (Note, all of the MATLAB code we used is publically available at: http://www.neuroeconlab.com/muse-data-collection.html). We found through pilot testing that if we could minimize the variance of a given channel (less than 150 uV^2^/s) then the number of lost trials was minimal (expect for blinks and other typical EEG artifacts, of course). Finally, we note that we deliberately chose a very short data collection window—our oddball task lasted 180 s - to emphasize the portability of our approach for field/clinical research. If we had of collected data for a longer period of time by extending the duration of both tasks by adding more trials then we would also have lost fewer participants in our post-experiment analysis of the EEG data.

### Event markers and marker timing

The ability to insert temporally accurate “markers” to yoke experimental stimuli to continuous EEG data has long been considered to be of critical importance when conducting ERP studies (Luck, 2014). Previous work with portable EEG systems has also sought to mark the data, either directly (Debener et al., 2012; Vos et al., 2014) or via temporal event synchronization (Wong et al., 2014). Here, as we noted previously, we chose not to mark the EEG data and instead we simply recorded segments of EEG data that were streamed from the MUSE to a laptop computer after each trial. Of course, we are aware that due to varying time lags inherent with the Bluetooth data connection and because we have a lack of experimental event markers there is considerable timing “jitter” in our EEG data (see Shorey and Miller, 2000). Specifically, one typically finds that Bluetooth has a lag of 18 to 20 ms with a jitter of approximately 5 ms (see Luque et al., 2012). However, our results clearly demonstrate that this does not matter if one simply wants to quantify ERP components—our data that we collected with the MUSE EEG system clearly revealed N200, P300, and reward positivity ERP components. To be fair, it is worth noting that our approach may be unsuitable for subtle within manipulations—further work is needed to address this concern.

Practically speaking, the random delays in the temporal onset of our data collection would have a Gaussian distribution and thus the lags would average out—indeed therefore our MUSE ERP components look so like the ones we observed with the reduced analysis. Further, while the timing onset via Bluetooth is not “guaranteed”—the order of data packets is (https://www.bluetooth.com/; Bray and Sturman, 2001), and as such, the averaging of temporal onset did not impact the present data as badly as we initially feared it might. Finally, we have written MATLAB code that allows two-way communication via the OSC protocol to send markers to mark a continuous EEG recording—however, these OSC markers would be susceptible to the same varying time lags as our direct recording protocol. In any case, the use of portable EEG systems does not preclude a protocol within which continuous EEG data is recorded and marked in a manner common to most ERP studies. However, as we have previously noted we deliberately sought here to move away from reliance on event-markers.

### Use of non-standard electrode locations

One consequence of using a portable EEG system such as the MUSE for ERP research is that the researcher has to accept that they will most likely be working with non-standard electrode locations for given ERP components. For example, for the two experimental tasks we employed here one would typically have focused offline analyses on electrodes Pz (the oddball task) and FCz (the reward-learning task) as opposed to on a pooled average of electrodes TP9 and TP10. However, we did not have electrodes at either Pz or FCz and as such we were forced to work with a pooled average of electrodes TP9 and TP10 for the oddball task and a pooled average of electrodes AF7 and AF8 for the reward-learning task.

With this in mind, the three ERP components that we observed (N200, P300, reward positivity) did not look like they typically do (see Figures 1–3)—but they were clearly observable and more importantly quantifiable in a reliable manner. We note here the importance that we were able to quantify the N200, P300, and reward positivity with a minimum number of trials with a portable MUSE EEG system—researchers are now able to measure these components quickly and reliably in the field or in clinical environments with relative ease. The ability to measure ERP components with a portable MUSE EEG system that we demonstrate here with a single computer and without the use of event-markers has the potential to greatly increase the use of EEG and ERPs as a clinical diagnostic tool and/or to allow greater use of EEG and ERPs in field research. In support of this, we note here that we recently utilized the MUSE EEG system to collect ERP data at Mount Everest Base Camp at an altitude of 17,598 feet (Krigolson and Binsted, in preparation) and in a hospital environment from medical students to assess fatigue (Howse, Walzak, Wright, and Krigolson, in preparation). Finally, we note that the MUSE system already has a large consumer base that has successfully used self-guiding software (i.e., the MUSE App). With this in mind, researchers could potentially program experiments in the iOS or Android software environments, distribute them via the respective App stores, and potentially collect EG/ERP data from thousands of participants.

## Conclusions

Improvements in the quality of low cost portable EEG systems such as the MUSE provide an excellent opportunity for researchers to improve their ability to conduct field and/or clinical research. Here, we demonstrate that we were able to quantify the N200, P300, and reward positivity ERP components with a MUSE EEG system in two experimental paradigms that together were completed in under 10 min. Our method utilized a single computer and we did not have to rely on the use of event-markers. We note here that all of the MATLAB code and protocols for researchers to replicate and extend our research are available on our laboratory website: http://www.neuroeconlab.com/muse.html.

## Ethics statement

University of Victoria HREB Written informed Consent.

## Author contributions

OK was the principle investigator for this project. FC was in charge of data collection and app development. AN and CH were in charge of programming and theoretical development of the project. CW was responsible for data analysis.

### Conflict of interest statement

The authors declare that the research was conducted in the absence of any commercial or financial relationships that could be construed as a potential conflict of interest.

## Footnotes

^1^We used a lower top end on the bandwidth filter as we expected the data from the MUSE to be noisier than that of our ActiChamp system. Further, the ERP components of interest here (N200, P300, reward positivity) are all characterized by a frequency composition of less than 15 Hz.

## Supplementary material

Supplementary Figure 1 **Bayesian histogram plots for all analyses (top: standard, middle: reduced, bottom: MUSE) and components (left: N200, middle: P300, right: reward positivity)**. Black bars represent 95% highest density intervals.
