# Supplementary figures and images for: Choosing MUSE: Validation of a Low-Cost, Portable EEG System for ERP Research
Source: Front Neurosci. 2017 Mar 10;11:109. doi: 10.3389/fnins.2017.00109 (PMC5344886; doi:10.3389/fnins.2017.00109)

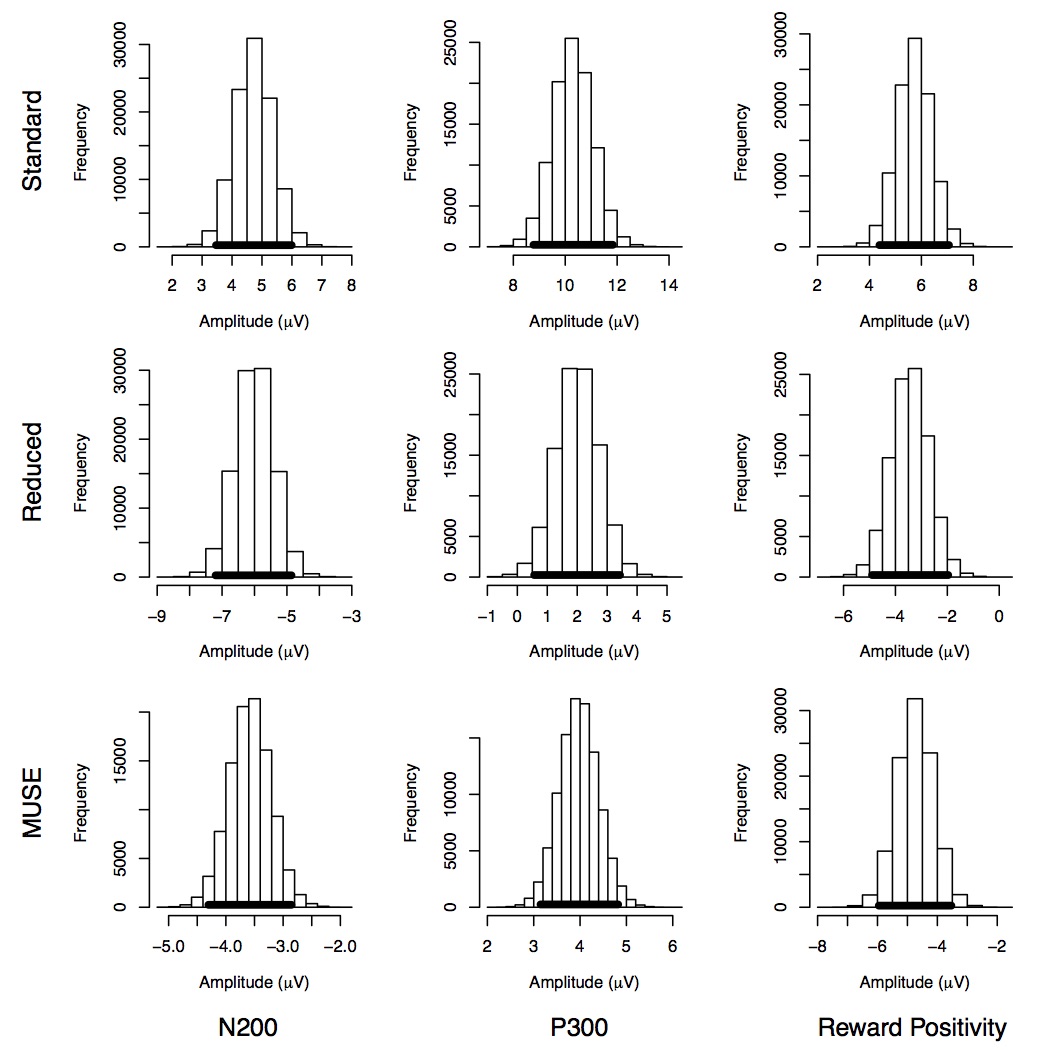

Supplement: Supplementary Figure 1 — Bayesian histogram plots for all analyses (top: standard, middle: reduced, bottom: MUSE) and components (left: N200, middle: P300, right: reward positivity). Black bars represent 95% highest density intervals. [file Image1.JPEG]
